# Metabolomics-guided analysis of isocoumarin production by *Streptomyces* species MBT76 and biotransformation of flavonoids and phenylpropanoids

**DOI:** 10.1007/s11306-016-1025-6

**Published:** 2016-03-30

**Authors:** Changsheng Wu, Hua Zhu, Gilles P. van Wezel, Young Hae Choi

**Affiliations:** Molecular Biotechnology, Institute of Biology, Leiden University, Sylviusweg, 72, 2333 BE Leiden, The Netherlands; Natural Products Laboratory, Institute of Biology, Leiden University, Sylviusweg, 72, 2333 BE Leiden, The Netherlands

**Keywords:** *Streptomyces*, Antibiotics, Growth phase-dependence, NMR-based metabolomics, Biotransformation

## Abstract

**Introduction:**

Actinomycetes produce the majority of the antibiotics currently in clinical use. The efficiency of antibiotic production is affected by multiple factors such as nutrients, pH, temperature and growth phase. Finding the optimal harvesting time is crucial for successful isolation of the desired bioactive metabolites from actinomycetes, but for this conventional chemical analysis has limitations due to the metabolic complexity.

**Objectives:**

This study explores the utility of NMR-based metabolomics for (1) optimizing fermentation time for the production of known and/or unknown bioactive compounds produced by actinomycetes; (2) elucidating the biosynthetic pathway for microbial natural products; and (3) facilitating the biotransformation of nature-abundant chemicals.

**Method:**

The aqueous culture broth of actinomycete *Streptomyces* sp. MBT76 was harvested every 24 h for 5 days and each broth was extracted by ethyl acetate. The extracts were analyzed by ^1^H NMR spectroscopy and the data were compared with principal component analysis (PCA) and orthogonal projection to latent structures (OPLS) analysis. Antimicrobial test were performed by agar diffusion assay.

**Results:**

The secondary metabolites production by *Streptomyces* sp. MBT76 was growth phase-dependent. Isocoumarins (**1**–**9**), undecylprodiginine (**10**), streptorubin B (**11**), 1*H*-pyrrole-2-carboxamide (**12**), acetyltryptamine (**13**), and fervenulin (**14**) were identified, and their optimal production time was determined in crude extracts without tedious chromatographic fractionation. Of these compounds, 5,6,7,8-tetramethoxyl-3-methyl-isocoumarin (**9**) is as a novel compound, which was most likely synthesized by a type I iterative polyketide synthase (PKS) encoded by the *icm* gene cluster. Multivariate data analysis of the ^1^H NMR spectra showed that acetyltryptamine (**13**) and *tri*-methoxylated isocoumarins (**7** and **8**) were the major determinants of antibiotic activity during later time points. The methoxylation was exploited to allow bioconversion of exogenously added genistein into a suite of methoxylated isoflavones (**15**–**18**). Methoxylation increased the antimicrobial efficacy of isocoumarins, but decreased that of the isoflavones.

**Conclusion:**

Our results show the applicability of NMR-based metabolic profiling to streamline microbial biotransformation and to determine the optimal harvesting time of actinomycetes for antibiotic production.

**Electronic supplementary material:**

The online version of this article (doi:10.1007/s11306-016-1025-6) contains supplementary material, which is available to authorized users.

## Introduction

The alarming increase of pathogen resistance underlines the urgent need for new antibiotics (WHO 2014). As producers of two-thirds of all known antibiotics and of many other bioactive compounds, actinomycetes are a rich source of clinical drugs, with the majority produced by members of the genus *Streptomyces* (Barka et al. 2016; Bérdy 2005). A problem is the high frequency of re-discovery of known compounds that has frustrated high-throughput (HT) screening regimes in the past decennia (Cooper and Shlaes 2011; Payne et al. 2007). Sequencing of the genomes of actinomycetes revealed that the potential of even the best-studied model organisms as producers of natural products have been grossly underestimated (Bentley et al. 2002; Cruz-Morales et al. 2013; Ikeda et al. 2003). However, many antibiotic biosynthetic gene clusters are poorly expressed under routine laboratory conditions. A new drug-discovery pipeline is rapidly gaining momentum, which is combining fluctuation of the (cryptic) antibiotics production with metabolic profiling-based identification of the bioactivity of interest (Scherlach and Hertweck 2009; Wu et al. 2015b; Zhu et al. 2014a).

In actinomycetes, the onset of antibiotic production typically coincides with the transition from vegetative to aerial growth in solid-grown cultures, which corresponds roughly to the onset of stationary phase in liquid-grown cultures (Bibb 2005; van Wezel and McDowall 2011). It is therefore very important to closely monitor the metabolic changes during the entire fermentation time, so as to optimize the fermentation for production of the bioactive molecules of interest. Conventional analytical methods, e.g. HPLC–UV, are often used to monitor the production of molecules but fail to provide structural information and the detection of analytes tends to be demanding on chromophores. To capture all the molecular features in a microbial mixture without prior information, techniques with a broader analytical capacity should be employed. Moreover, to take a snapshot of multiple cellular processes at for example any given time-point of a growth curve of a producing microorganism, a robust analytical platform is needed, which should be rapid, reproducible, stable in time, and simple in sample preparation (Kim et al. 2011). Proton nuclear magnetic resonance (^1^H NMR) spectroscopy provides both qualitative (chemical shift and splitting pattern) and quantifiable (integrative area) peaks for structure elucidation (sometimes aided by 2D NMR), allowing tracing of metabolic changes over time by visual comparison of the metabolomes harvested at several time-points (Novoa-Carballal et al. 2011). Subtle metabolic differences could be further highlighted by unbiased statistical filtering of the ^1^H NMR spectra, such as chemometric methods, principle component analysis (PCA), partial least squares discriminant analysis (PLS-DA), *etcetera*.

Actinomycetes do not only producing a plethora of natural products, but they can also efficiently perform metabolic conversions on molecules with a range of physicochemical properties (Wu et al. 2015d). Deciphering the metabolic tailoring reactions of natural products produced by a certain producing strain over time, such as glycosylation, hydroxylation, *O*-methylation, or prenylation (Medema et al. 2015), will provide insights into the optimal time of providing starter molecules in biotransformation experiments, since many enzymes show significant promiscuity in terms of substrate specificity (Schwab 2003).

In this study, NMR-based metabolomics was used to determine the variation in metabolites produced by *Streptomyces* sp. MBT76 at five different time-points and correlated these to the observed bioactivity using multivariate data analysis (MVDA). Aided by UPLC-ToF–MS analysis, various bioactive molecules (**1**–**14**) including a new *tetra*-methoxylated isocoumarin (**9**), were thus identified in the culture broth. Biotransformation of the isoflavone genistein by MBT76 led to a group of methoxylated derivatives **15–18**. Methoxylation increased the efficacy of isocoumarins against both Gram-positive and Gram-negative bacteria, while conversely, methoxylation decreased the antimicrobial efficacy of isoflavones.

## Materials and methods

### Culture media and condition

*Streptomyces sp.* MBT76 was obtained from the culture collection of Molecular Biotechnology, IBL, Leiden University. Liquid Minimal Medium (NMMP) (Hodgson 1982) was used as medium for fermentation. Fully supplemented NMMP contained per liter 2 g (NH_4_)_2_SO_4_, 5 g casaminoacids, 0.6 g MgSO_4_.7H_2_O, 1 ml minor elements solution, 15 mM Na^+^–K^+^ phosphate buffer pH 6.8, 0.5 % mannitol, 1 % glycerol and 8 g peptone. Spores of *Streptomyces* sp. MBT76 were inoculated into 50 ml of culture medium, and cultures were incubated at 30 °C with continuous shaking at 220 rpm.

### Isolation of bioactive compounds

*Streptomyces* sp. MBT76 mycelia were removed by centrifugation at 4,500×*g* for 10 min. The spent media (culture supernatant) was extracted twice with 20 ml of ethyl acetate. The organic phase was washed with 30 ml of distilled water and dried with 5 g of anhydrous Na_2_SO_4_. Finally, the ethyl acetate solvent was evaporated under vacuum at 40 °C and the resultant dried material was dissolved in 1.5~2.0 ml of ethyl acetate in a microtube (Eppendorf type-5415C, Hamburg, Germany). The crude extract was evaporated under room temperature to remove organic solvent, and subsequently dipped into liquid nitrogen and lyophilized by freeze drier (Edwards Ltd., Crawley, UK). The obtained residue was independently dissolved in methanol-*d*_4_ for ^1^H NMR analysis (see below). All experiments were conducted in five replicates.

After ^1^H NMR measurement, the five 96 h samples were combined and dissolved in methanol (50 ml), which was defatted twice with 20 ml of *n*-hexane. The resulting crude extract was directly separated by semi-preparative HPLC, which was performed with a Agilent 1200 series HPLC apparatus (Agilent technologies Inc, Santa Clara, CA, USA) equipped with a reversed-phase C_18_ column (Phenomenex Luna C18 (2) 100 Å 5 micron 250 × 10 mm, Torrance, CA, USA). The separation was eluted with a gradient of acetonitrile in water from 20 % to 70 % in 50 min at 2 ml/min flow rate, to obtain compounds **1** (*t*_R_ = 31.52 min, 1.14 mg), **3** (*t*_R_ = 35.75 min, 1.62 mg), **7** (*t*_R_ = 38.34 min, 0.34 mg), **8** (*t*_R_ = 39.50 min, 0.28 mg).

### NMR measurement and data analysis

NMR sample preparation and measurements were done according to our previously published protocol (Kim et al. 2010). Briefly, 0.5 ml of methanol-*d*_4_ was added to the freeze-dried sample. Subsequently, the mixture was vortexed for 10 s and sonicated for 20 min at 42 kHz using an Ultrasonicator 5510E-MT (Branson, Danbury, CT, USA), followed by centrifugation at 13,000×*g* at room temperature for 5 min. The supernatant (0.3 ml) was transferred to a 5 mm micro NMR tube and analyzed. The ^1^H NMR spectra were recorded at 25 °C on a 600 MHz Bruker DMX-600 spectrometer (Bruker, Karlsruhe, Germany) operating at a proton NMR frequency of 600.13 MHz. Deuterated methanol was used as the internal lock. Each ^1^H NMR spectrum consisted of 128 scans using the following parameters: 0.16 Hz/point, pulse width (PW) = 30 (11.3 μs) and relaxation delay (RD) = 1.5 s. Free induction decays (FIDs) were Fourier transformed with a line broadening (LB) = 0.3 Hz. The resulting spectra were manually phased and baseline corrected, and calibrated to residual methanol-*d*_4_ at 3.30 ppm, using XWIN NMR (version 3.5, Bruker). The ^1^H NMR data files were processed as described by Kim et al. (2010). The AMIX software (Bruker Biospin GmbH) was used to reduce the ^1^H NMR spectra to an ASCII file, with total intensity scaling. Bucketing or binning was performed and the spectral data were reduced to included regions of equal width (0.04 ppm) equivalent to the region of *δ* 0.30–10.00. The regions of *δ* 4.85–4.95 and 3.25–3.35 were removed in the analysis because of the remaining signal of solvents of HDO and CD_3_OD, respectively. Principal component analysis (PCA) and orthogonal projection to latent structures (OPLS) based on Pareto scaling were performed with the SIMCA-P+ software (version 13.0, Umetrics, Umeå, Sweden).

### UPLC-ToF–MS analysis

UPLC-ToF–MS analyses were performed on an UPLC system (Ultimate 3000, ThermoScientific, Germany) coupled to an ESI-llQ-ToF spectrometer (micrOToF-QII, Bruker Daltonics, Germany) in the positive mode. The chromatographic separation was done using a Kinetex C_18_ UPLC 2.6 µm particle size column 150 × 2.0 mm (Phenomenex, Torrance, CA, USA) at a flow rate of 0.3 ml/min and a column temperature of 30 °C. Samples (3 µl) were eluted using a gradient of solvent A (water) and B (acetonitrile), both with 0.1 % formic acid (v/v). The initial percentage of B was 5 %, which was linearly increased to 90 % in 19.5 min, followed by a 2 min isocratic period and, then re-equilibrated with original conditions in 2 min. Nitrogen was used as drying and nebulizing gas. The gas flow was set at 10.0 l/min at 250 °C and the nebulizer pressure was 2.0 bar. The MS data were acquired over *m/z* range of 100–1000. The capillary voltage was 3.5 kV. For internal calibration, a 10 mM solution of sodium formate (Fluka, Steinheim, Germany) was infused. Formic acid, water and acetonitrile were LCMS grade, Optima (Fisher Scientific, Waltham, MA,USA).

### Biotransformation

Substrates (10 mg) dissolved in 200 μl DMSO were fed to 2-day old *Streptomyces* sp. MBT76 culture broth. This biotransformation period lasted for another 3 days. The control experiment included both negative control of feeding 200 μl DMSO solvent to MBT76, and positive control of 10 mg substrates dissolved in 200 μl DMSO added to sterile culture media. Each experiment was done in triplicate. The method used to harvest the biotransformation products was as described for the metabolomics study.

To isolate the biotransformation products of genistein (Sigma), the combined mixture in 50 ml methanol was first defatted twice with 20 ml of *n*-hexane, which was subsequently fractionated by Sephadex LH-20 chromatography (GE Healthcare Life Sciences, Eindhoven, The Netherlands) eluting with methanol, to give seven fractions fr1–7. These fractions were subjected to NMR profiling to discard fr1–3, and fr6 that gave no isoflavone signals. Fr7 was purified by silica gel (pore size 60 Å, 70–230 mesh, St. Louis, MO, USA) to yield compound **15** (0.7 mg). Fr4 was separated by preparative TLC on a silica gel plate (Si60, Merck, Darmstadt, Germany), migrated with solvent system of CHCl_3_-MeOH (20:3) to give pure compound **16** (0.5 mg) and semi-pure compound **18** (0.2 mg). Fr5 was purified by preparative TLC to give pure compound **17** (0.3 mg) using the same TLC conditions.

### Antimicrobial activity of the isocoumarins and isoflavones

Antimicrobial test were performed by agar diffusion assay, against the Gram-positive indicator strain *Bacillus subtilis* 168 and the Gram-negative *Escherichia coli* K12, as we previously described (Wu et al. 2015c). The respective compounds were dissolved in methanol (2 mg/ml), and 20 μl of the solution was applied on a paper disc (6 mm diameter). The discs were then placed onto an agar plate containing a soft agar overlay with either *Bacillus subtilis* 168 or *Escherichia coli* K12. After incubation at 37 °C for 18 h, growth inhibition zones (in mm) were monitored visually as a measure of the antimicrobial activity.

### Genome sequencing, assembly, and annotation

Genome sequencing and annotation was done as described previously (Girard et al. 2014). Illumina/Solexa sequencing on Genome Analyzer IIx and sequencing on PacBio RS were outsourced to BaseClear BV (Leiden, The Netherlands). 100-nt paired-end reads were obtained and the quality of the short reads verified using FastQC (http://www.bioinformatics.bbsrc.ac.uk/projects/fastqc/). Depending on quality, reads were trimmed to various lengths at both ends. Processed raw reads were subsequently used as input for the Velvet assembly algorithm. Genomes were annotated using the RAST server with default options. Contigs were also annotated using GeneMark.hmm for ORF prediction, BLASTP for putative function prediction and HMMER for protein-domain prediction, manually inspected for some and visualized using Artemis. *Streptomyces* sp. MBT76 was subjected to Illumina/Solexa whole genomic sequencing, and the genome was assembled in 13 contigs, with a total genome size of 8.64 Mb. In total 7974 coding sequences (CDS) were predicted. Analysis of the contigs by AntiSMASH (Blin et al. 2013) presented a possible 55 putative biosynthetic gene clusters (BGCs) specifying secondary metabolites. The genome has been deposited at GenBank under the accession LNBE00000000.

## Results and discussion

### Correlated growth phase with chemotype

*Streptomyces* sp. MBT76 was previously identified as a prolific producer of antibiotics, including two highly methoxylated isocoumarins (Zhu et al. 2014b). To assess the correlation between growth and antibiotic production, MBT76 was grown in the liquid minimal medium (NMMP) supplemented with 0.8 % (w/v) peptone, which has a positive effect on the strain’s antibiotic producing capacity (Zhu et al. 2014b). Samples for the determination of biomass (dry weight) and antibiotic production (measured as zones of inhibition against indicator strains) were collected at 24 h time intervals until 120 h. A long lag phase of growth was observed that covered the first 24 h, after which the strain grew exponentially from 24–50 h. After around 80 h, the culture entered a phase where biomass decreased rapidly, most likely due to nutrient exhaustion (Figure S1, A). Antibiotic activity against *Bacillus subtilis* 168 was not seen before 48 h (Figure S1, B), and the onset of antibiotic production was seen after around 60 h of growth. The largest inhibition zone (25 mm) was found for supernatants obtained after 96 h, when cultures had already entered the death phase.

**Fig. 1:**
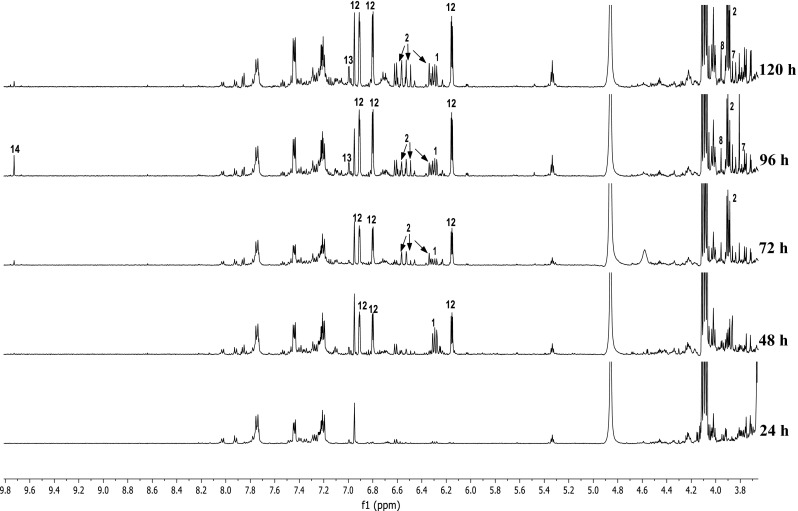
NMR profiling of *Streptomyces* sp. MBT76 harvested at 24-h intervals. The ^1^H NMR spectra varied significantly in aromatic region *δ* 6.0–8.0, and methoxyls region *δ* 3.45–4.25, with more complex proton signals as time progressed. Assigned signals are numbered as summarized in Table 1

**Fig. 2:**
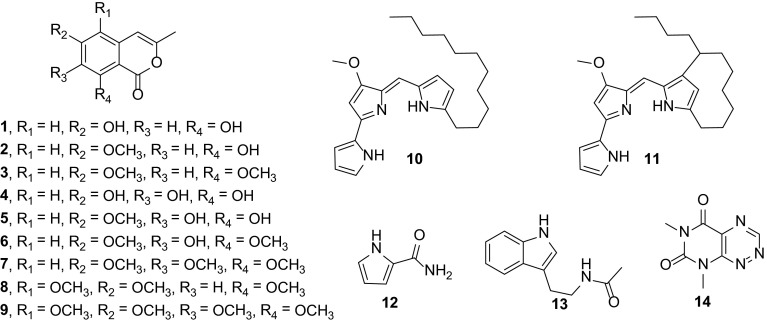
Secondary metabolites produced by *Streptomyces* sp. MBT76

NMR-based metabolomics was employed to correlate bacterial growth and antimicrobial activity with the corresponding metabolic changes over time. At five time-points, namely after 24, 48, 72, 96, and 120 h of inoculation, the biomass of MBT76 was removed by centrifugation and the compounds present in the spent media were extracted with ethyl acetate. The obtained metabolomes were subjected to ^1^H NMR profiling and antimicrobial assay against *B. subtilis* 168. The ^1^H NMR spectra varied significantly in aromatic region *δ* 6.0–8.0, and methoxyls region *δ* 3.45–4.25, with more complex proton signals as time progressed (Fig. 1). Low levels of secondary metabolites were produced before 48 h, while 72 h appeared to be a turning point in terms of secondary metabolites production. 96 h samples exhibited the most complex metabolome as compared to the other groups, coinciding with the highest antimicrobial activity against *B. subtilis.* The compounds in the crude extracts were elucidated with ^1^H NMR spectra and UPLC-ToF–MS analysis (retention time, UV spectrum, and high resolution mass). The metabolites included methoxylated isocoumarins (**1**–**9**) (Zhu et al. 2014b); red-pigmented undecylprodiginine (**10**) and streptorubin B (**11**) (Cerdeño et al. 2001); 1*H*-pyrrole-2-carboxamide (**12**) (Salem et al. 2014); acetyltryptamine (**13**) (Mehdi et al. 2009); and fervenulin (**14**) (Ruanpanun et al. 2011) (Fig. 2; Table 1). Among these, the highly methoxylated compound 5,6,7,8-tetramethoxy-3-methyl-isocoumarin (**9**) has not been previously described. This novel molecule was determined based on the similarity of its UV spectrum with those of congeners **1**–**8** and a high resolution mass at *m/z* [M + H]^+^ of 281.1039, calculated molecular formula C_14_H_17_O_6_ (Figure S2).

**Table 1:** Spectral data assignments for the compounds displayed in Fig. 2

NO	Compounds	Characteristic^1^H NMR chemical shifts (ppm)	Experimental mass (Da)	UV λ_max_ (nm)
1	6,8-Dihydroxy-3-methyl-isocoumarin	6.29 (*d*, *J* = 1.2, 1H); 6.24 (d, *J* = 2.4, 1H); 6.27 (*d*, *J* = 2.4, 1H); 2.21 (*d*, *J* = 1.2, 3H)	193.0511 [M + H]^+^; 215.0312 [M + Na]^+^	244, 328
2	6-Methoxyl-8-hydroxy-3-methyl-isocoumarin		207.0662 [M + H]^+^; 229.0478 [M + Na]^+^; 435.1053 [2 M + Na]^+^	244, 326
3	6,8-Dimethoxyl-3-methyl-isocoumarin	6.29 (*d*, *J* = 1.2, 1H); 6.47 (*d*, *J* = 2.4, 1H); 6.52 (*d*, *J* = 2.4, 1H); 2.23 (*d*, *J* = 1.2, 3H); 3.89 (s, 3H); 3.90 (s, 3H)	221.0830 [M + H]^+^; 243.0646 [M + Na]^+^	244, 328
4	6,7,8-Trihydroxy-3-methyl-isocoumarin		209.0441 [M + H]^+^	244, 328
5	6-Methoxyl-7,8-dihydroxy-3-methyl-isocoumarin	6.57 (*d*, *J* = 1.2, 1H); 6.38 (s, 1H); 3.75 (s, 3H); 2.26 (s, 3H)	223.0612 [M + H]^+^; 245.0424 [M + Na]^+^	244, 340
6	6,8-Dimethoxy-7-hydroxy-methyl-isocoumarin		237.0779 [M + H]^+^; 259.0593 [M + Na]^+^; 495.1256 [2 M + Na]^+^	240, 340
7	6,7,8-trimethoxy-3-methyl -isocoumarin	6.35 (*d*, *J* = 1.2, 1H); 6.81 (s, 1H); 2.22 (*d*, *J* = 1.2); 3.95 (s, 3H); 3.83 (s, 3H); 3.90 (s, 3H)	251.0935 [M + H]^+^; 273.0756 [M + Na]^+^; 523.1596 [2 M + Na]^+^	246, 336
8	5,6,8-Trimethoxy-3-methyl -isocoumarin	6.55 (*d*, *J* = 1.2, 1H); 6.70 (s, 1H); 2.20 (*d*, *J* = 1.2, 3H); 3.76 (s, 3H); 4.00 (s); 3.95 (s, 3H)	251.0935 [M + H]^+^; 273.0756 [M + Na]^+^; 523.1596 [2 M + Na]^+^	246, 336
9	5,6,7,8-Tetramethoxy-3-methyl -isocoumarin		281.1039 [M + H]^+^; 303.0861 [M + Na]^+^; 583.1804 [2 M + Na]^+^	240, 337
10	Undecylprodiginine	7.23 (s, 1H); 6.56 (s, 1H); 6.46 (dd, *J* = 4.8, 3.0, 1H); 6.39 (d, *J* = 4.8, 1H); 4.10 (s, 3H); 2.35 (t, *J* = 7.2, 2H)	394.2857 [M + H]^+^	
11	Streptorubin B	6.73 (s, 1H); 6.53 (s, 1H); 6.44 (dd, *J* = 4.8, 3.0, 1H); 6.41 (*d*, *J* = 4.8, 1H); 4.11 (s, 3H)	392.2692 [M + H]^+^	
12	1*H*-pyrrole-2-carboxamide	6.92 (dd, *J* = 2.4, 1.2, 1H); 6.81 (dd, *J* = 3.6, 1.2, 1H); 6.17 (t, *J* = 3.6, 2.4, 1H)	111.0562 [M + H]^+^; 133.0372 [M + Na]^+^	268
13	Acetyltryptamine	7.54 (dd, *J* = 7.8, 1.2, 1H); 7.31 (brd, *J* = 8.4, 1H); 7.70 (td, *J* = 7.8, 1.2, 1H); 6.99 (td, *J* = 7.8, 1.2, 1H); 7.05 (s, 1H); 3.45 (t, *J* = 7.2, 2H); 2.56 (*t*, *J* = 7.2, 2H); 1.90 (s, 3H)	203.1211 [M + H]^+^; 225.1011 [M + Na]^+^	224, 282
14	Fervenulin	9.72 (s, 1H); 3.80 (s, 3H); 3.47 (s, 3H);	194.0705 [M + H]^+^; 216.0482 [M + Na]^+^	240, 344

Compound identification was based on ^1^H NMR, high resolution mass spectrometry, and UV absorption spectrum. Proton coupling constants (*J* in Hz) are given in parentheses

**Fig. 3:**
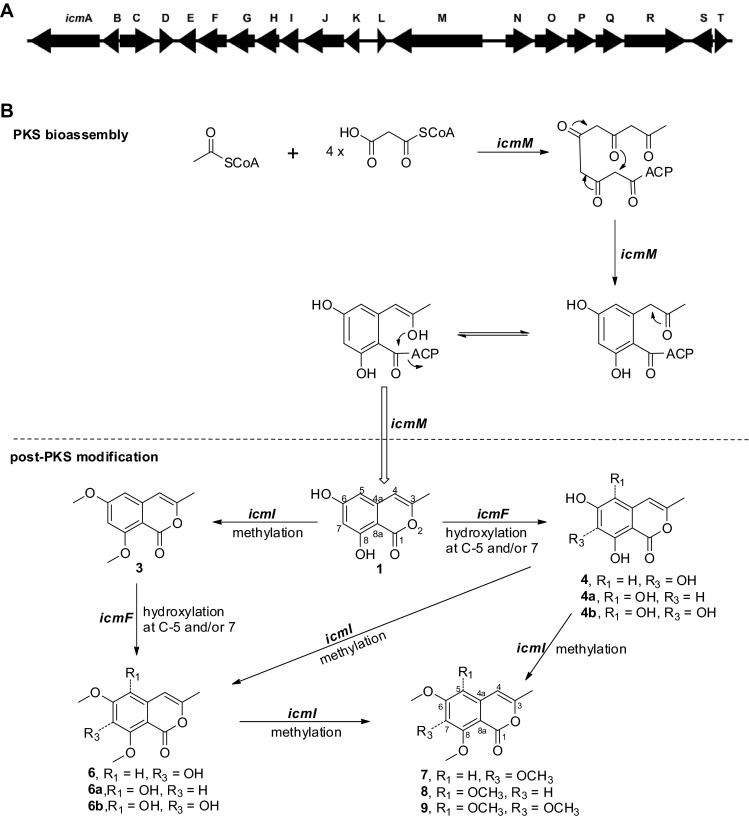
Biosynthetic pathway of isocoumarins produced by *Streptomyces* sp. MBT76. **a** Organization of the type I iterative PKS gene cluster (*icm*) in *Streptomyces* sp. MBT76 (Table S1). **b** Proposed biosynthetic route to highly methoxylated isocoumarins **7–9**. The modular non-reducing PKS gene *icmM* was iteratively used to assemble the basic backbone 6,8-dihydroxy-3-methyl-isocoumarin (**1**), which was further furnished into a range of methoxylated isocoumarins by the oxidoreductase *icmF* and the methyltransferase *icmI* in the post-PKS stage

A plausible biosynthetic pathway (Fig. 3) is proposed for isocoumarins **1**–**9**. Although isocoumarin polyketides are frequently reported in actinomycetes (Singh et al. 2013; Zinad et al. 2011), the underlying genetic basis is poorly understood. In view of the biosynthesis of the isocoumarin backbone in fungi (Cochrane et al. 2016; Ishiuchi et al. 2012; Nakazawa et al. 2012; Zaehle et al. 2014), along with dihydroisocoumarin assembly in the actinomycete *Saccharopolyspora erythraea* (Sun et al. 2012) and in sponge-associated bacteria (Fisch et al. 2009), we tentatively propose that isocoumarin biosynthesis in *Streptomyces* sp. MBT76 is executed by a type I iterative modular polyketide synthase (PKS). Indeed, antiSMASH (Blin et al. 2013) analysis of the genome of *Streptomyces* sp. MBT76 identified a gene cluster (*icm*, Table S1) that encodes the candidate PKS components. This contains the *icmM* gene that encodes multiple catalytic domains including ketosynthase (KS), acyl transferase (AT), dehydrase (DH), acyl carrier protein (ACP), which is likely responsible for the assembly of the precursor 6,8-dihydroxy-3-methyl-isocoumarin (**1**). This low-order isocoumarin could be further tailored by the oxygenase encoded by *icmF*, and an *O*-methyltransferase encoded by *icmI*.

The coexistence of compounds **7**, **8**, and **9** confirmed all the non-bridged carbons (positions 5, 6, 7, and 8) in benzene ring of isocoumarin could be methoxylated. This suggests that MBT76 could produce more new isocoumarin-type intermediates than those that have been elucidated here, like **4a**, **4b**, **6a**, and **6b**. Because both C-5 and C-7 of **1** could be hydroxylated, the substituted pattern (5,6,8-trihydroxylation or 6,7,8-trihydroxylation) in compounds **5** and **6** remained unclear. Some ambiguity remained regarding the *mono*- and/or *di*- methylation patterns in compounds **2**, **5**, and **6**, as all four–OH groups on ring A could be methylated. Further up-scale fermentation followed by extensive phytochemical isolation is needed to clarify ambiguities in structures of **2**, **5**, and **6**, and to unveil other new derivatives of isocoumarin family.

**Fig. 4:**
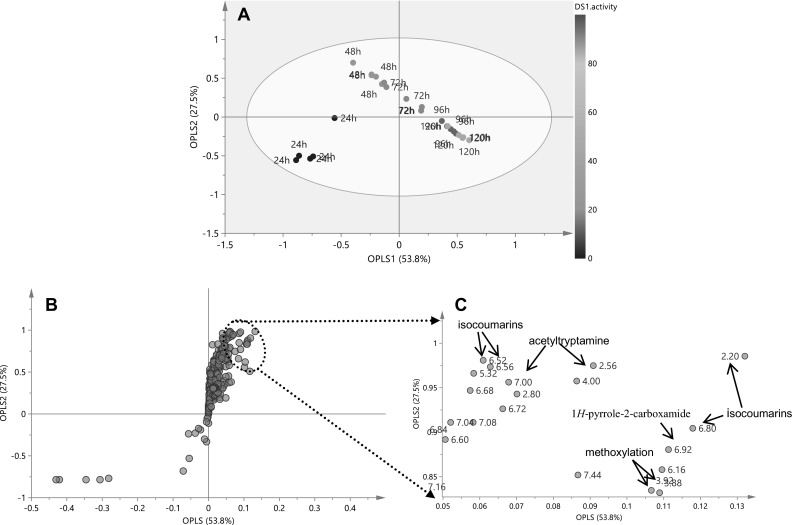
Supervised multivariate data analysis of the NMR spectra. **a** orthogonal projection to latent structures (OPLS) *score plot* showed separation of different time-points along OPLS1, and **b** the corresponding *S*-*plot* highlighted the compounds responsible for better activity in latter time points (96 and 120 h). **c** These compounds were identified as acetyltryptamine (**13**), and methoxylated isocoumarins (**7** and **8**) as annotated. *Y* value in OPLS analysis was antimicrobial bioactivity against *Bacillus subtilis* 168

Multivariate data analysis (MVDA) was utilized to correlate the antimicrobial activity with the responsible signals in the ^1^H NMR spectra. An unsupervised MVDA, principal component analysis (PCA) was first used to discriminate samples, but in this way only the non-active 24 h group was separated from other time-points (data not shown). As a next step to correlate metabolites detected in the ^1^H NMR spectra with the bioactivity, orthogonal projection to latent structures (OPLS) was applied to the data using Y-variable of antimicrobial activity (inhibition percentage), allowing the removal of spectral X-variables unrelated to the sample classes chosen and filtration of the biomarkers from the biological varieties (Trygg and Wold 2003). The regression has been validated using cross validation analysis of variance (CV-ANOVA), with a *P* value <0.001. Separation of all the harvesting times could be observed in the OPLS score plot, with the active group (72, 96, and 120 h) clustered in the positive side along the OPLS1 axis (Fig. 4a). The *S*-plot presented the signals contributable to the discrimination among the samples with diversified antibacterial activities. The major signals responsible for the bioactivity were assigned as 6,7,8-trimethoxyl-3-methyl-isocoumarin (**7**), 5,7,8-trimethoxyl-3-methyl-isocoumarin (**8**), and acetyltryptamine (**13**) (Figs. 4, b, c). The antibiotic activity may at least in part be attributed to acetyltryptamine (**13**), which was previously reported to have broad-spectrum antibiotic properties (Mehdi et al. 2009). To test whether methoxylation helped increase the antimicrobial activity of isocoumarins, we isolated four major isocoumarins (**1**, **3**, **7**, and **8**) with low and/or high level of methoxylation. Comparison of the antimicrobial activity of these compounds was performed by agar diffusion assay, against the Gram-positive *B. subtilis* 168 and the Gram-negative *E. coli* K12 (Figure S3). The *tri*-methoxylated isocoumarins **7** and **8** exhibited pronounced inhibition of growth of *B. subtilis*, while compounds **1** and **3** only gave negligible inhibition zones. The same trend was also observed for inhibition against *E. coli*, though the bioactivity of the compounds against this strain was much lower than against *B. subtilis*. This result validated the OPLS analysis that attributed *tri*-methoxylated isocoumarins **7** and **8** rather than non-methoxylated precursor **1** as the discriminators for the higher bioactivity at 96 h and 120 h. Based on this observation, we anticipated that the new compound 5,6,7,8-tetramethoxyl-3-methyl-isocoumarin (**9**) should have even better antimicrobial property than **7** and **8**, but its purification failed due to intrinsic low abundance (Figure S2). To correlate harvesting time to specific metabolites, we used time as the *Y*-variable in the OPLS analysis, resulting in a similar separation pattern of the five time-points along OPLS1 (Figure S4, a). The major discriminators for 96 h and 120 h groups were the same to those for activity as *Y*-variable OPLS analysis (Figure S4, b). Taken together, the dynamic ^1^H NMR profiling and MVDA demonstrated the time-dependent biosynthesis of antibiotics (**1**–**14**) produced by *Streptomyces* sp. MBT76. This strain began to produce precursor 6,8-dihydroxy-3-methyl-isocoumarin (**1**) at 48 h, whose methoxylation into **2**–**9** at all the non-bridged carbons of the benzene ring occurred after 72 h, whereby the highly methoxylated isocoumarins **7** and **8** peaked at 96 h. Prodiginines (**10** and **11**) were observed throughout growth, which was reflected by the abundant by-product 1*H*-pyrrole-2-carboxamide (**12**) initiated at 48 h and the red appearance of the culture broth (Cerdeño et al. 2001). The antibiotics acetyltryptamine (**13**) and fervenulin (**14**) were particularly produced at later time-points.

### Metabolomics-driven biotransformation of phenylpropanoids

**Fig. 5:**
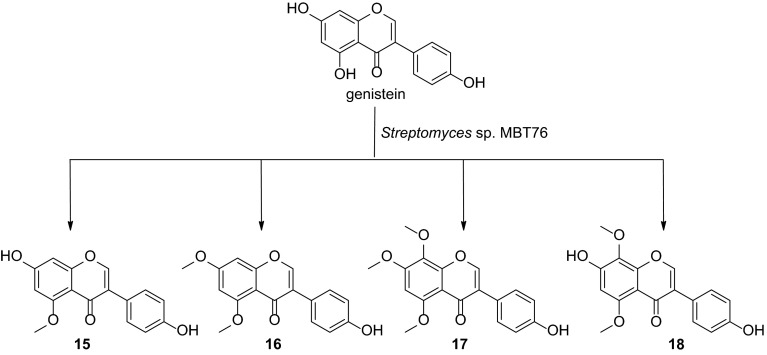
Biotransformation of genistein by S*treptomyces* sp. MBT76. Products were identified on the basis of NMR and/or UPLC-ToF–MS high resolution mass

Inspired by the post-PKS methoxylation of 6,8-dihydroxy-3-methyl-isocoumarin (**1**, Fig. 3), we then wanted to see what would happen in a biotransformation experiment whereby the isoflavone genistein was fed to cultures of *Streptomyces* sp. MBT76. Genistein and other isoflavones contain a similar fused benzopyranone moiety as isocoumarins and the same *meta*-hydroxylation on ring A. In accordance with the dynamic metabolism of isocoumarins as shown by the NMR-based metabolomics, genistein was fed to MBT76 culture broth at 48 h, *i.e.* in the ‘late exponential’ growth phase, and incubation was allowed to continue for another 72 h. The ethyl acetate extract was then subjected to NMR and TLC analysis and compared to control samples. To identify genistein-derived biotransformation products, we performed NMR-guided chromatographic separation (Wu et al. 2015a) by tracking the downfield aromatic singlet (H-2 around *δ* 8.0) that is characteristic of isoflavones in the ^1^H NMR spectrum. The purified products were subsequently subjected to NMR for structure elucidation. The ^1^H NMR (methanol-*d*_4_, 600 MHz) spectrum of compound **16** presented two methoxyls, besides two *meta*-coupled aromatic doublets, which allowed identification of one of the genistein-derived biotransformation products as 4′-hydroxy-5,7-dimethoxy-isoflavone (**16**) (Miyazawa et al. 2006). In comparison, the major discrepancy in the ^1^H NMR spectrum of compound **15** was a single *O*-CH_3_, which indicated that a *mono*-methylation had occurred in the ring A. When DMSO-*d*_6_ was used as solvent, the downfield-shifted exchangeable hydroxyl *δ* 11.5–13.5 that originates from the intramolecular hydrogen bonding with ketone (C-4) had disappeared, which was indicative of methylation at the 5-OH. Thus, compound **15** was identified as 4′,7-dihydroxy-5-methoxy-isoflavone. The ^1^H NMR spectrum of compound **17** identified three methoxyls at *δ* 3.94, 4.02, and 3.85, together with one uncoupled singlet at *δ* 6.68, which indicated *tri*-methoxylation at ring A. The possibility of methylation on ring B (4′-OH) was ruled out by HMBC and HSQC NMR. Furthermore, the two correlations H-6/5-OCH_3_ and H-6/7-OCH_3_ in the ^1^H–^1^H NOESY spectrum confirmed that compound **17** was 4′-hydroxy-5,7,8-trimethoxy-isoflavone. Compound **18** shared the same substitution mode with **17** in ring A because of one uncoupled singlet at *δ* 6.48, but it presented two methoxyls in the ^1^H NMR spectrum. One methyl was located at 8–OH based on the HMBC correlation from 8-OCH_3_ (*δ* 3.86) to C–8 (*δ*_C_ 134.4). The other methyl was located at the 5-OH rather than at the 7-OH because no signal was observed in the range of *δ* 11.5–13.5 when measured in DMSO-*d*_6_. Taken together, genistein underwent exactly the same methylation pattern of isocoumarin **1**, as summarized in Fig. 5. In consideration of the structural similarity between isocoumarin **1** and genistein, it is conceivable that the same *O*-methyltransferase (*icmI*) is involved in the methylation of both substrates. Interestingly, this *O*-methyltransferase methylated isoflavone at all the three hydroxyl groups on ring A, but not at the single *p*-hydroxyl group on ring B.

Motivated by the biotransformation of genistein, we also investigated whether the structurally related flavone apigenin and the dihydroflavonol naringenin could also be methoxylated. Unexpectedly, both apigenin and naringenin resisted biotransformation by MBT76, as evidenced by TLC and NMR analysis. The linkage pattern of the benzene ring B on chromone (at either C-2 or C-3) might influence the efficiency of methylation. From the perspective of biosynthesis it is interesting to note that in plants, ring B of flavonoids originates from cinnamic acid (Park et al. 2009). Therefore, we further tested whether hydroxycinnamic acids could be methylated. Six commercially available congeners, including cinnamic acid, *o*, *m*, *p*- coumaric acid, caffeic acid, and ferulic acid, were fed to the *Streptomyces* sp. MBT76. The corresponding biotransformation products were identified in mixture through ^1^H NMR profiling and confirmed by UPLC-ToF–MS analysis. Amidation at the carboxyl group was the exclusive modification for all the (hydroxyl)cinnamic acids, with the exception of ferulic acid that could also be methylated at the 4-OH group (Figure S5). Unlike the situation of isocoumarins, where activity increases on methylation, methylated isoflavones had lower bioactivity than the unmethylated precursors. As shown in Figure S3, genistein showed highest antibiotic activity against both *B. subtilis* and *E. coli*, while *tri*-methoxylated biotransformation product **19** failed to inhibit growth of the indicator strains. This observation was in accordance with previous results that the free phenolic OH groups of flavonoids are crucial for potent antimicrobial activity against Gram-positive and Gram-negative bacteria (Thongnest et al. 2013).

The in vitro biochemical reaction may in some cases be more advantageous than in vivo biotransformation for functional characterization of enzymes, e.g. due to limited uptake of the substrate, or because substrates may have an effect on overall gene expression of the various biosynthetic pathways. While the expected in vivo methylation of apigenin, naringenin, and most (hydroxyl)cinnamic acids was not observed, the potential promiscuity of methyltransferase IcmI suggested by post-PKS modification of isocoumarin (Fig. 3) and biotransformation of genistein (Fig. 5) is intriguing and may allow the methylation of a broader range of chemical skeletons. This is currently being investigated in our laboratory.

## Conclusion

The growth phase has a major influence on the production of secondary metabolites in *Streptomyces* sp. MBT76. Four days of incubation was optimal to accumulate antimicrobial compounds identified as 6,7,8-trimethoxyl-3-methyl-isocoumarin (**7**), 5,7,8-trimethoxyl-3-methyl-isocoumarin (**8**), 5,6,7,8-tetramethoxyl-3-methyl-isocoumarin (**9**), and acetyltryptamine (**13**). However, production of 6,8-dihydroxyl-3-methyl-isocoumarin (**1**) was optimal after two days of incubation. NMR spectroscopy in conjugation with multivariate data analysis effectively identified specialized metabolites in the crude extract of MBT76, but also highlighted differences between the metabolomes at the different time-points. The methodology applied in this work should be useful for scientists who seek to identify the optimal fermentation time to harvest desirable bioactive compounds from cultures of in principle any microorganism that produces promising bioactive molecules, such as those with potential industrial or medical application.

## Electronic supplementary material

Below is the link to the electronic supplementary material.

Supplementary material 1 (DOCX 1392 kb)
