# Supplementary material for: Metabolomics-guided analysis of isocoumarin production by Streptomyces species MBT76 and biotransformation of flavonoids and phenylpropanoids
Source: Metabolomics. 2016 Mar 30;12:90. doi: 10.1007/s11306-016-1025-6 (PMC4819732; doi:10.1007/s11306-016-1025-6)
Supplement: Supplementary file 1 — Supplementary material 1 (DOCX 1392 kb) [file 11306_2016_1025_MOESM1_ESM.docx]

**Metabolomics-guided analysis of isocoumarin production by *Streptomyces* species MBT76 and biotransformation of flavonoids and phenylpropanoids**

Changsheng Wu ^1, 2,^ ^, Hua Zhu ^1,^ ^, Gilles P. van Wezel ^1,*^, and Young Hae Choi ^2,*^

^1^ *Molecular Biotechnology, Institute of Biology, Leiden University, Sylviusweg 72, 2333 BE, The Netherlands*

^2^ *Natural Products Laboratory, Institute of Biology, Leiden University, Sylviusweg 72, 2333 BE, The Netherlands*

^ These authors contributed equally to this work

* Corresponding author. Young Hae Choi: Tel. +31 715274510, y.choi@chem.leidenuniv.nl; Gilles P. van Wezel: Tel +31 715274310; g.wezel@biology.leidenuniv.nl

**Table S1. Gene organization of type I iterative non-reducing PKS gene cluster (*icm*) for isocoumarins biosynthesis in *Streptomyces* sp. MBT76.**

| **ORF** | **Product** | **Accession in GeneBank** | **Length** | **Putative function** | **Homologue in *Streptomyces roseoverticillatus*** | **Homology** |
| --- | --- | --- | --- | --- | --- | --- |
| 1 | *icm*A | WP_058043254.1 | 943 | LuxR family transcriptional regulator | WP_030365549.1 | 84% |
| 2 | *icm*B | WP_058043255.1 | 224 | TetR family transcriptional regulator | WP_043188202.1 | 92% |
| 3 | *icm*C | WP_058043256.1 | 496 | MFS transporter | WP_043188355.1 | 84% |
| 4 | *icm*D | WP_058043257.1 | 200 | NADPH-dependent FMN reductase | WP_030365552.1 | 88% |
| 5 | *icm*E | WP_058043258.1 | 246 | ToxA protein | WP_030365553.1 | 87% |
| 6 | *icm*F | WP_058043259.1 | 416 | oxidoreductase | WP_052392495.1 | 71% |
| 7 | *icm*G | WP_058043260.1 | 376 | diaminohydroxyphosphoribosylaminopyrimidine deaminase/5-amino-6-(5-phosphoribosylamino)uracil reductase | WP_030365554.1 | 88% |
| 8 | *icm*H | WP_058043261.1 | 330 | Serine/threonine kinase | WP_030365555.1 | 90% |
| 9 | *icm*I | WP_058043262.1 | 251 | methyltransferase type 12 | WP_043188204.1 | 81% |
| 10 | *icm*J | WP_058043263.1 | 570 | WD repeat-containing protein | WP_030365558.1 | 95% |
| 11 | *icm*K | WP_058043264.1 | 208 | GTP cyclohydrolase II | WP_043188207.1 | 98% |
| 12 | *icm*L | WP_058043265.1 | 130 | Hypothetical protein | WP_030365560.1 | 88% |
| 13 | *icm*M | WP_058043266.1 | 1239 | iterative type I polyketide synthase | WP_030365561.1 | 91% |
| 14 | *icm*N | WP_058043267.1 | 397 | AMP-dependent synthetase/ligase | WP_030365562.1 | 95% |
| 15 | *icm*O | WP_058043268.1 | 434 | MFS transporter | WP_053688342.1 | 81% |
| 16 | *icm*P | WP_058043269.1 | 381 | hypothetical protein | WP_030365564.1 | 93% |
| 17 | *icm*Q | WP_058043270.1 | 388 | acyl-protein synthase | WP_030365565.1 | 96% |
| 18 | *icm*R | WP_058043271.1 | 834 | hypothetical protein | WP_030365566.1 | 94% |
| 19 | *icm*S | WP_058043272.1 | 303 | phosphotriesterase | WP_052392497.1 | 88% |
| 20 | *icm*T | WP_058043273.1 | 188 | Zn-ribbon-like motif containing protein | WP_030365568.1 | 88% |

**A**

**B**

**Figure S1. Growth curve of *Streptomyces* sp. MBT76 (A) and corresponding antimicrobial activity against *Bacillus subtilis* (B).** Some 1.5 ml culture was collected every two hours during the daytime from the first to the fifth inoculation day. Separated supernatant from the pellet by centrifuging and kept them independently. The pellet was heated at 70 °C for overnight to determine the dry weight, while the supernatant was accessed for antimicrobial activity against *Bacillus subtilis* 168. The data were means of three replicates.

**[M + H]^+^**

**[M + Na]^+^**

**[2M +Na]^+^**

**D**

**C**

**B**

**A**

**9**

**3**

**7**

**1**

**Figure S2. U(H)PLC-UV-ToF-HRMS analysis of *Streptomyces* sp. MBT76 crude extract. A**) UV chromatogram of *Streptomyces* sp. MBT76 crude extract, whereby the isocoumarins **1**, **3**, **7**, **9** were labelled; **B**) UV spectrum of 6,8-dimethoxyl-3-methyl-isocoumarin (**3**); **C**) UV spectrum of 5,6,7,8-tetramethoxy-3-methyl-isocoumarin (**9**); **D**) HRESIMS spectrum of 5,6,7,8-tetramethoxy-3-methyl-isocoumarin (**9**); Electrospray (ESI) detection was achieved in positive ion mode.

**1**

**3**

**7**

**8**

**
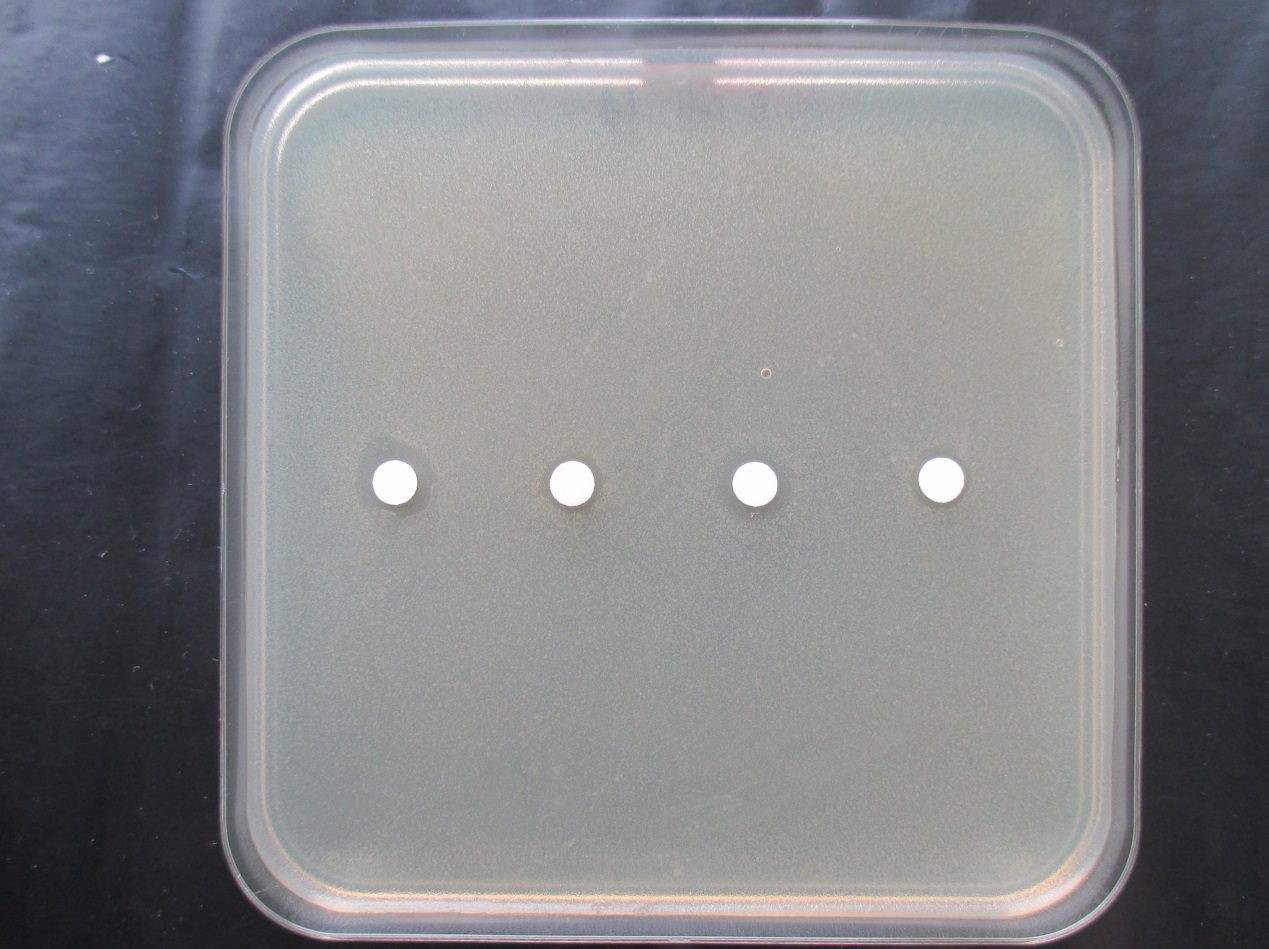

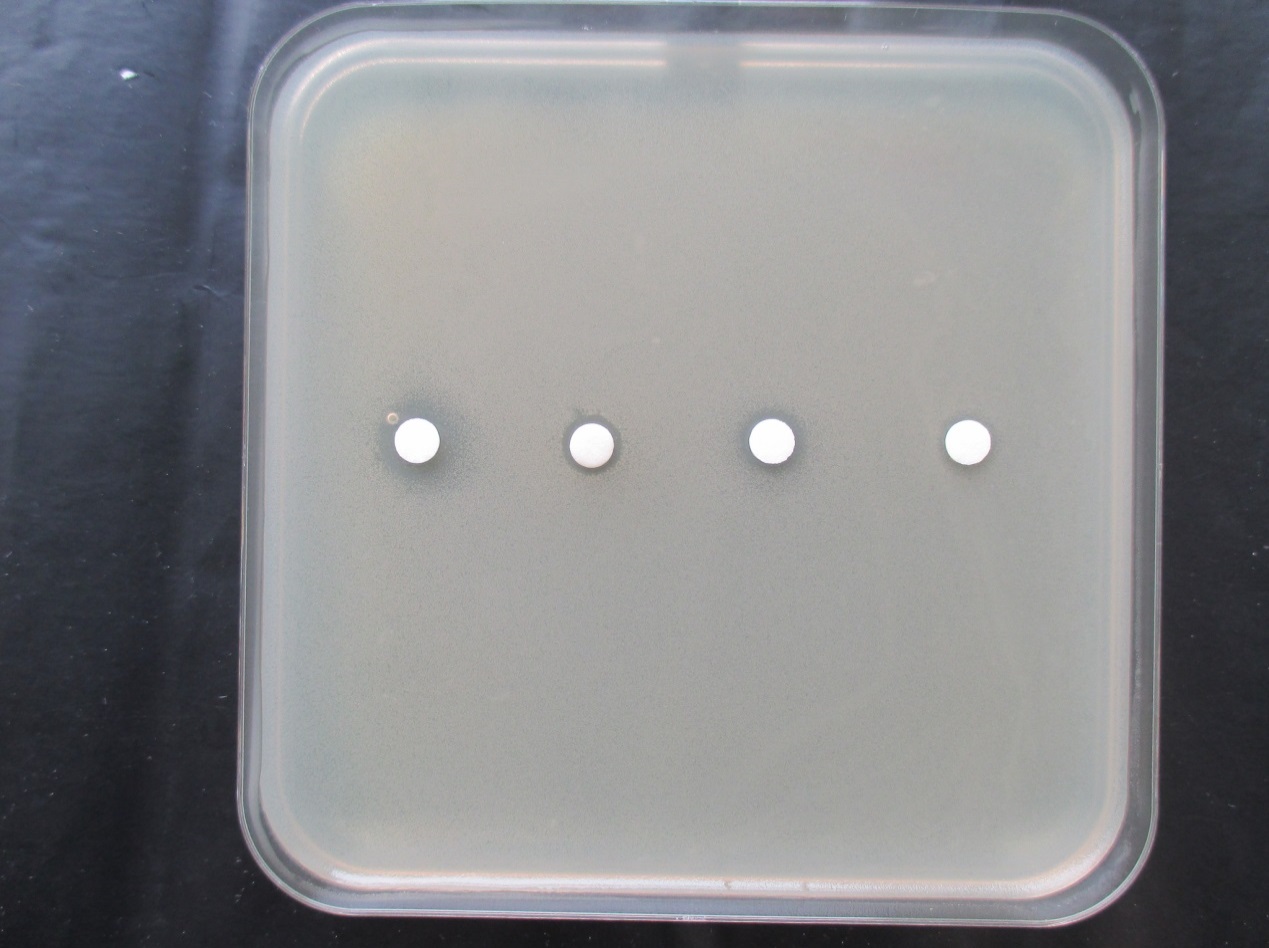

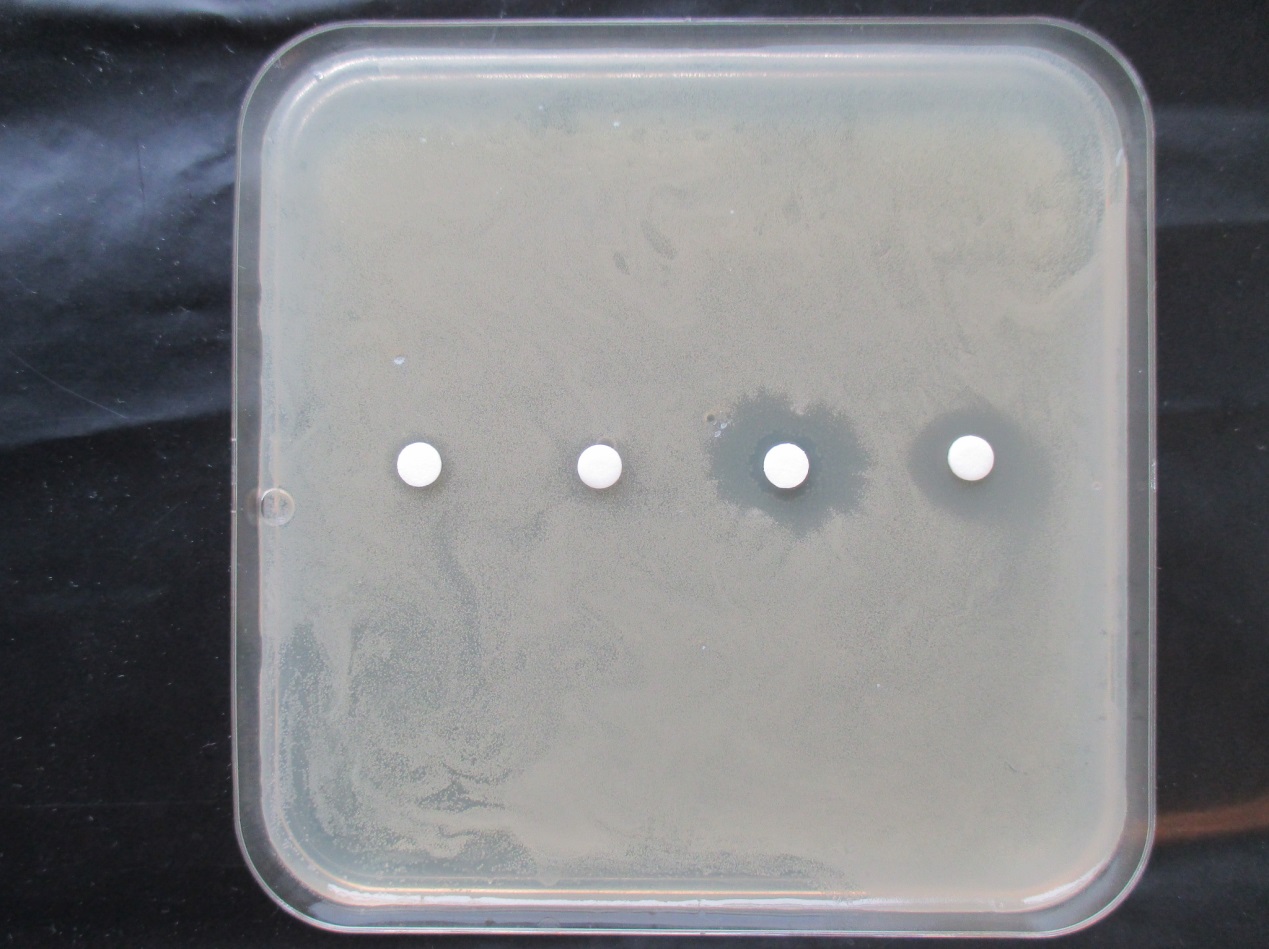
**

**17**

**16**

**15**

**genistein**

*E. coli*

*B. subtilis*

*B. subtilis*

*E. coli*

**
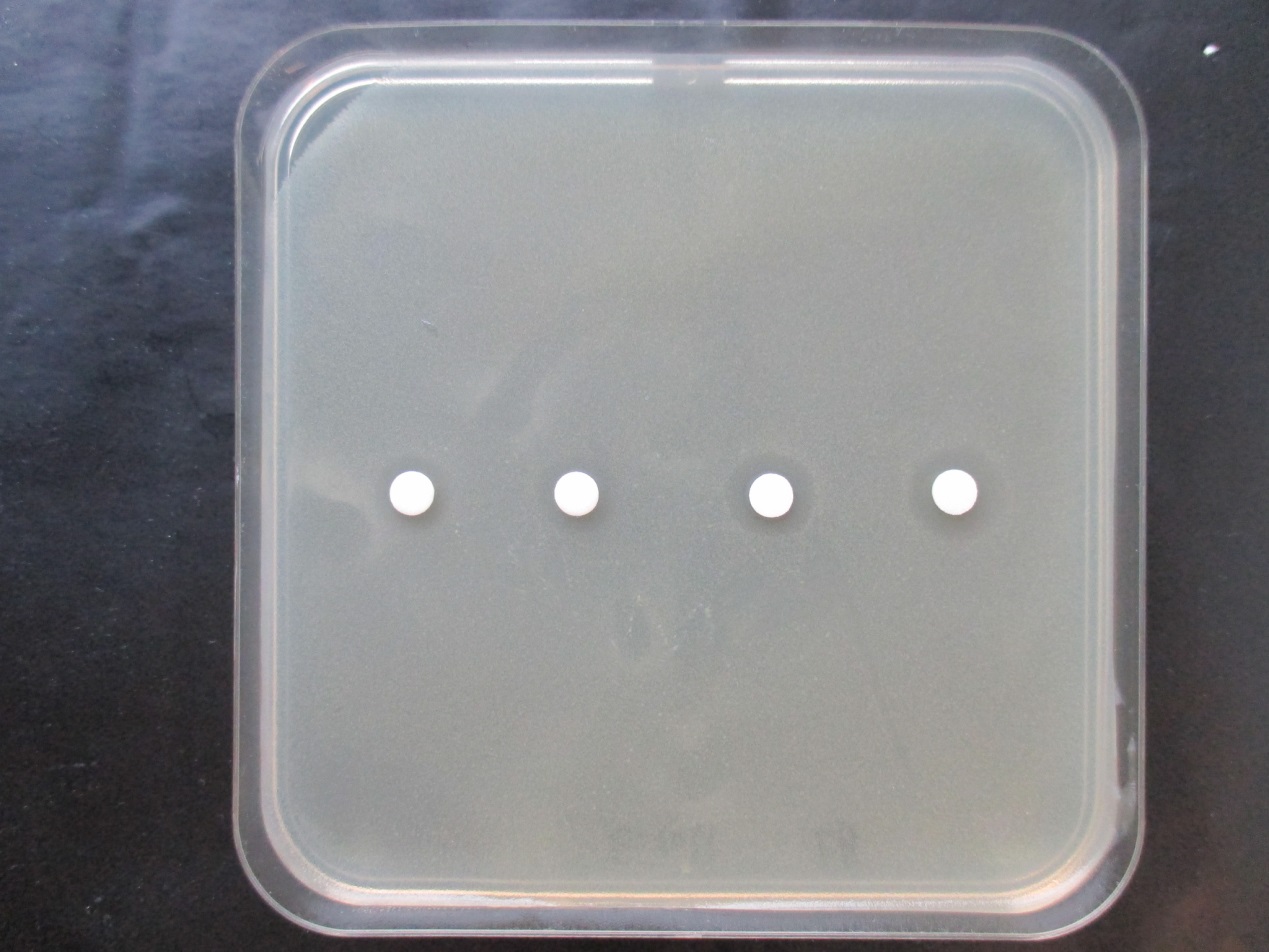
**

**Figure S3. Antimicrobial activity of selected isocoumarins and isoflavones.** The methoxylation enhanced the antimicrobial potency of isocoumarins against both Gram-positive *Bacillus subtilis* 168 and Gram-negative *Escherichia coli* K12, which in turn validated the metabolomics OPLS analysis in Figure 4. On the contrary, methylation decreased the antimicrobial activity of isoflavones.

**A**

**B**

**Figure S4. OPLS analysis of the NMR spectra by using time as the Y-variable.** (A) score plot, and (B) the corresponding loading S-plot.

**Figure S5. Biotransformation study in S*treptomyces* sp. MBT76 by using apigenin, naringenin, and (hydroxyl)cinnamic acids as substrates.** Products were identified on the basis of ^1^H NMR and/or UHPLC-TOF-MS high resolution mass.
